# An updated systematic review and Meta-analysis of the prevalence of type 2 diabetes in Iran, 1996–2023

**DOI:** 10.3389/fpubh.2024.1322072

**Published:** 2024-04-04

**Authors:** Narjes Hazar, Mohammad Jokar, Negin Namavari, Saeed Hosseini, Vahid Rahmanian

**Affiliations:** ^1^Diabetes Research Center, Shahid Sadoughi University of Medical Sciences, Yazd, Iran; ^2^Young Researchers and Elite Club, Islamic Azad University of Karaj, Karaj, Iran; ^3^Research Center for Non Communicable Disease, Jahrom University of Medical Sciences, Jahrom, Fars, Iran; ^4^Center for Healthcare Data Modeling, Department of Biostatistics and Epidemiology, School of Public Health, Shahid Sadoughi University of Medical Sciences, Yazd, Iran; ^5^Department of Public Health, Torbat Jam Faculty of Medical Science, Torbat Jam, Iran

**Keywords:** epidemiology, diabetes mellitus, prevalence, meta-analysis, Iran

## Abstract

**Background:**

Diabetes mellitus (DM) poses a significant threat to public health, and the anticipated surge of over 100% in the age-standardized prevalence of type 2 diabetes in Iran between 2021 and 2050 underscores the pressing need for focused attention. The rationale for estimating the prevalence of type 2 diabetes in Iran becomes even more compelling when considering the potential cascading effects on the healthcare system, quality of life, and economic burden. The aim of this study was to estimate the prevalence and trends of DM from 1996 to 2023 in the Islamic Republic of Iran.

**Methods:**

Up to July 2023, without deadlines, the search for appropriate articles in Persian and English. Iranian sources including SID, Magiran, and Element were included in the databases, along with foreign ones like PubMed/MEDLINE, Web of Science, Science Direct, Embase, Scopus, ProQuest, and Google Scholar. Using the JBI quality checklist, the study’s level of quality was evaluated. Version 14 of STATA was used to carry out the statistical analysis. The Dersimonian and Liard random-effects models were used because of heterogeneity. To investigate the causes of heterogeneity, subgroup analysis and univariate meta-regression were utilized. Sensitivity analysis was then carried out to see how each study’s findings affected the final findings. The prevalence pattern over time was also followed using cumulative meta-analysis.

**Results:**

There were 53 studies in all, with a combined sample size of 1,244,896 people. Men were predicted to have a type 2 diabetes prevalence of 10.80% (95% CI: 9.1–12.4), while women were assessed to have a prevalence of 13.4% (95% CI: 11.6–15.3). Additionally, the prevalence of diabetes was much higher in the 55–64 age group, coming in at 21.7% (95% CI: 17.5–25.0). The anticipated prevalence of diabetes was 7.08% for 1988 to 2002, 9.05% for 2003 to 2007, 9.14% for 2008 to 2012, 15.0% for 2013 to 2017, and 13.40% for 2018 to 2023, among other time periods. Geographically, type 2 diabetes was most prevalent in Khuzestan (15.3%), followed by Razavi Khorasan (14.4%), Qazvin (14.3%), and Yazd (12.6%).

**Conclusion:**

The prevalence of type 2 diabetes was estimated at 10.8%, highlighting variations across gender, age groups, and geographic regions that underscore the necessity for specific interventions. These findings advocate for proactive measures, including tailored screening and lifestyle modification programs. The notable temporal increase from 2013 to 2017 signals the need for policymakers and healthcare practitioners to develop effective strategies, anticipating and addressing the potential future burden on the healthcare system.

**Systematic Review Registration:**

https://www.crd.york.ac.uk/prospero/display_record.php?ID=CRD42023437506, identifier: CRD42023437506.

## 1 Background

Diabetes is recognized as the primary cause of morbidity and mortality worldwide (1). It is a chronic and complex disease. According to estimates from the Global Burden of Disease study, diabetes was the eighth leading cause of mortality and disability worldwide in 2019, affecting nearly 460 million people (2) across all countries and age groups. The resulting medical expenses have placed a heavy pressure on healthcare systems. 537 million people worldwide are estimated to have diabetes according to predictions from the International Diabetes Federation from 2021 (3), with global healthcare spending expected to exceed $1,054 billion by 2045.The possibility of slowing the rise in diabetes prevalence by 2025 is less than 1% for women and significantly lower for males, despite worldwide efforts to manage and prevent the disease (4). While type 2 diabetes is characterized by a non-autoimmune progressive loss of adequate-cell insulin secretion against a background of metabolic syndrome and insulin resistance, type 1 diabetes is recognized by the American Diabetes Association (ADA) 2023 classification system as an autoimmune condition that results in pancreatic-cell destruction and complete insulin deficiency. Notably, the conventional categorization of diabetes type 1 as exclusively affecting children and type 2 as only affecting adults is no longer accurate (5). Age over 40, obesity, high blood pressure, an abnormal lipid profile, a lack of physical exercise, a history of gestational diabetes, smoking, and alcohol use are all risk factors for type 2 diabetes. Amputation of the lower limbs, blindness, retinopathy, nephropathy, and atherosclerotic cardiovascular disease are only a few of the serious consequences and organ damage that diabetic patients run the risk of (6).

The age-standardized global prevalence of type 2 diabetes is expected to surge significantly, increasing by 61.2% from 5.9% in 2021 to 9.5% in 2050, impacting a staggering 1.27 billion individuals worldwide. This prevalence exhibits notable variations across super-regions, ranging from 82.7% in North Africa and the Middle East to 30.3% in the high-income region. Moreover, the age-standardized prevalence of type 2 diabetes is projected to more than double in specific countries in North Africa and the Middle East between 2021 and 2050. These nations include Oman, United Arab Emirates, Syria, Iran, Libya, Sudan, and Saudi Arabia (7).

Various statistics on the prevalence of diabetes in Iran are available. A meta-analysis study conducted between 1996 and 2004 indicated that 24% of those over 40 had diabetes (8). Lower prevalence rates, however, have been found by more recent research. The prevalence of diabetes was found to be 26.1% in Yazd in 2015 (28% in women and 22.9% in men) (9), and 14.4% in Tehran’s population in 2017 (10). According to a study from the World Health Organization from 2020, Iran’s total prevalence of diabetes was 10.3%, with estimates for men and women of 9.6 and 11.1%, respectively. Obesity (24.9%), physical inactivity (31.9%), and overweight (60.5%) were the main risk factors for this illness in Iran. In light of anticipated surge in total diabetes prevalence, there is a compelling need to undertake a comprehensive analysis to assess and estimate the prevalence of diabetes in Iran. Such an analysis is vital for gaining insights into the specific challenges faced by Iran and for developing targeted strategies to address the growing impact of diabetes within the country. The aim of this study was to estimate the prevalence and trends of DM from 1996 to 2023 in the Islamic Republic of Iran.

## 2 Methods

### 2.1 Protocol and registration

According to the PRISMA-P 2020 guidelines for reporting items in systematic reviews and meta-analysis protocols, this systematic review and meta-analysis adhered to the process set out in that document. The study’s protocol was entered into the PROSPERO database and given the registration number CRD42023437506. The details can be accessed at: https://www.crd.york.ac.uk/prospero/#recordDetails.

### 2.2 Search strategy

**Figure. 1:**
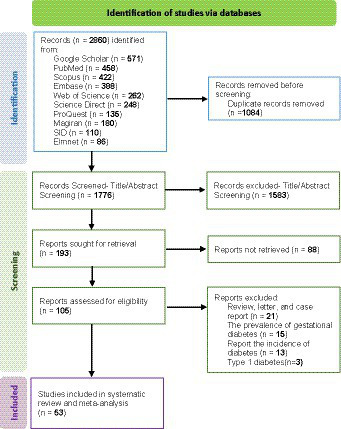
PRISMA flowchart of studies included in this systematic review and meta-analysis.

The researchers conducted a time-unrestricted search for pertinent articles in Iranian databases including SID (Scientific Information Database), Magiran, and Element, as well as international databases such as PubMed/MEDLINE, Web of Science, ScienceDirect, Embase, Scopus, ProQuest, and Google Scholar from the inception of their investigation until July 2023. The search strategy utilized a combination of relevant keywords and controlled vocabulary specific to each database (Supplementary Table 1). In Iranian databases, Persian terms and their English equivalents were used, while in international databases, English-language terms were employed. The search strategy included both Persian and English-language publications. Boolean operators “AND” and “OR” were utilized to combine search terms effectively. Additionally, reference lists of identified articles were manually searched to ensure comprehensive coverage of relevant literature. The PRISMA flowchart (Figure 1) depicts the process of identifying and selecting relevant publications.

### 2.3 Eligibility criteria

Studies required to be original full-text papers published in peer-reviewed journals up to July 2023, in either English or Persian, and done in Iranian settings in order to be included in this systematic review and meta-analysis. Furthermore, this systematic review and meta-analysis included cross-sectional studies that reported the prevalence of diabetes. Exclusion criteria were employed to filter out articles that did not adhere to specific requirements. Notably, non-observational studies, including short communications and review articles, were excluded. Additionally, studies with smaller sample sizes, non-random sampling techniques, and those involving a mixture of type 2 and type 1 diabetes were not considered for inclusion.

### 2.4 Study risk of bias assessment

Using the JBI quality assessment technique for prevalence studies (11), two reviewers provided a critical evaluation of the included papers. Nine characteristics make up this instrument, which evaluates many factors like suitable sampling, concise research descriptions, sufficient sample sizes, reliable procedures and measurements, and acceptable statistical analysis. For each parameter, a score of 1 was given for a “Yes” response and a score of 0 for a “No” or “Unclear” response. Each article’s mean score was calculated. The identity of the journal and the authors’ identities were not withheld from the reviewers as they independently assessed the research. The reliability value for the Cohen’s Kappa test, which was used to measure the inter-rater agreement, was 0.838 (*p* < 0.001), suggesting very good agreement between the two reviewers. The total possible score for each paper ranged from 0 to 9. Based on their overall scores, the studies were divided into three categories: low risk of bias (8, 9), moderate risk of bias (4–7), and high risk of bias (0–3). Conflicts between reviewers were resolved through consensus discussion. In cases where discrepancies arose in the assessment of individual studies, the two reviewers discussed their interpretations and reasons for scoring each criterion. If consensus could not be reached, a third independent reviewer was consulted to adjudicate the disagreement.

### 2.5 Selection process

This research employed a multi-step procedure to extract accurate data. All chosen articles were imported into EndNote X8, and duplicates were eliminated. Then, the two team members independently examined the remaining articles’ titles and abstracts to eliminate any irrelevant studies. The criteria for selection were based on reports pertinent to the research topic and consistent with methods of analytical study. The World Health Organization (WHO) criteria were used for diagnosing diabetes in this research (5).

### 2.6 Data extraction

During the data extraction process, the included studies’ pertinent data were retrieved, including the author (s)’ names, publication year, sample size, total positive number, location, age group, gender, study design, BMI, and prevalence. In cases where discrepancies arose between the two team members during data extraction, a consensus was reached through discussion and re-evaluation of the respective articles. If disagreements persisted, a third senior researcher was consulted to provide an impartial resolution.

### 2.7 Synthesis methods

STATA 14 was used for the statistical analysis in this meta-analysis. Using inverse variance and Cochran Q statistics, the degrees of heterogeneity among the included studies were assessed. Low, moderate, and high levels of heterogeneity were determined, respectively, by I^2^ values of less than 50%, between 50 and 80%, and higher than 80%. The Dersimonian and Liard random-effects models were used for the statistical analysis due to the heterogeneity. To learn more about the causes of heterogeneity, several methods were used, including subgroup analysis and univariate meta-regression. The One-out-remove method was used in the sensitivity analysis to determine the effect of each research on the final outcomes. Using Egger’s regression, publication bias was evaluated. The total estimate was modified using the trim-and-fill approach in order to account for the estimated number of studies that were lost to censorship (12). The trend of changes in prevalence over time was examined using a cumulative meta-analysis test. Using the ArcGIS 10.3 program, the regional distribution of type 2 diabetes prevalence throughout Iranian provinces was examined.

## 3 Results

### 3.1 Study selection

Based on the inclusion criteria, 2,860 articles from the available databases were chosen. Then, 1776 articles with repetitive titles and abstracts were further examined after 1,084 duplicate entries were eliminated. One hundred ninety three papers were eliminated after a full-text examination, leaving 107 publications to be evaluated for eligibility. The systematic review and meta-analysis comprised 53 papers in total (Figure 1).

### 3.2 Study characteristics

The features of the selected studies are presented in detail in Table 1. 53 studies in all were selected, 21 of which were done between 1988 and 2007 and 32 between 2013 and 2023.

**Table 1:** Description of the studies included in the meta-analysis.

First author	Years	District	Total number	Total positive number	Prevalence %	Male prevalence (%)	Female prevalence (%)	QA
Veghari et al. (13)	2010	Golestan	1999	165	8.3	NR	NR	5
Azizi et al. (14)	2003	Iran	595,717	21,637	3.6	2.6	4.3	4
Shirani et al. (15)	2009	Isfahan	12,514	700	5.6	NR	NR	7
Ostovaneh et al. (16)	2014	Amol	5,826	716	10.7	NR	NR	6
Ostovaneh et al. (16)	2014	Zahedan	2,243	155	5.2	NR	NR	6
Lotfi et al. (17)	2014	Yazd	14,993	178	11.9	9.2	25.3	7
Khalilzadeh et al. (9)	2015	Yazd	403	105	26.1	22.9	28	6
Javadi et al. (18)	2009	Tehran	7,989	759	9.5	NR	NR	7
Hadaegh et al. (19)	2008	Tehran	9,489	877	9.1	8.7	6.3	7
Esteghamati et al. (20)	2009	Iran	5,287	460	8.7	NR	NR	8
Esteghamati et al. (21)	2006	Tehran	514	154	30	26.4	36.4	5
Saberi et al. (22)	2011	Kashan	429	30	7	7	NR	4
Azimi-Nezhad et al. (23)	2008	Razavi Khorasan	3,778	207	5.5	5.1	5.8	7
Larigani et al. (24)	2004	Tehran	1,573	171	10.9	8.9	12.2	6
Sadeghi et al. (25)	2007	Isfahan	12,514	789	6.3	5.4	7.1	7
Firozabadi et al. (26)	2002	Fars	384	16	4.2	4.68	3.64	4
Hadaegh et al. (27)	2008	Tehran	9,519	866	9.1	9	9.4	6
Aboutorabi et al. (28)	2012	Razavi Khorasan	210	28	13.3	NR	NR	5
Merac et al. (29)	2012	Isfahan	3,000	198	6.6	4.86	8.27	6
Saffari et al. (30)	2010	Razavi Khorasan	100	20	20	12.69	32.43	5
Dehghani et al. (31)	2023	Yazd	9,747	1747	17.9	15.39	20.46	7
Rahmanian et al. (32)	2013	Fars	892	103	11.6	11.1	12.1	6
Amini et al. (33)	2007	Isfahan	2,368	243	10.3	12.9	9.4	6
Mirzaei et al. (34)	2020	Yazd	9,965	1,378	14.1	12.4	15.6	7
Hariri et al. (35)	2021	Khuzestan	30,498	4,673	15.3	15.27	15.34	7
Khamseh et al.(36)	2021	Iran	163,770	24,565	15	NR	NR	6
Barati et al. (37)	2021	Tehran	2,123	303	14.3	13.5	15	8
Tanjani et al. (38)	2014	Iran	1,350	297	22	18.84	24.85	6
Esteghamati et al. (39)	2008	Iran	89,400	6,883	7.7	7.1	8.3	6
Sharifi et al. (40)	1999	Zanjan	1977	56	2.83	NR	NR	5
Larijani et al. (41)	2002	Bushehr	982	69	7.02	5.64	7.8	5
Larijani et al. (41)	1998	Bushehr	1,036	130	12.58	NR	NR	5
Gharipour et al. (42)	2003	Isfahan	800	52	6.5	NR	NR	4
Afkhamiardakani et al. (43)	2002	Yazd	2,795	387	13.84	14.15	13.6	5
Azizi et al. (44)	2003	Tehran	1,603	192	12	12	17	7
Azizi et al. (45)	2002	Tehran	6,899	372	5.4	5	5.7	6
Fakhrzadeh et al. (46)	2002	Bushehr	1,255	133	10.6	10.6		4
Larijani et al. (47)	2003	Qazvin	950	136	14.31	15.9	12.7	6
Salem et al. (48)	2003	Kerman	756	112	14.7	8.6	19.1	7
Navaee et al. (49)	2002	Iran	2033	150	7.37	NR	NR	5
Saadat et al. (50)	2002	Tehran	9,229	553	6	NR	NR	5
Shamsoldin et al. (51)	2002	Kerman	125	11	8.8	NR	NR	4
Sanjari et al. (52)	2007	Tehran	4,480	12	0.27	NR	NR	5
Khodakarami et al. (53)	2016	Iran	89,404	7,510	8.4	7.6	9.2	7
Khodakarami et al. (53)	2004	Iran	29,991	2,700	9	8.3	9.7	7
Khodakarami et al. (53)	2007	Iran	12,103	1,343	11.1	9.7	12.5	7
Khodakarami et al. (53)	2011	Iran	30,541	4,031	13.2	11.5	14.6	7
Oraii et al. (54)	2022	Tehran	8,151	1,504	18.5	18.7	18.2	8
Moradpour et al. (55)	2022	Iran	3,996	400	10	8.68	11.02	7
Hosseinpanah et al. (56)	2007	Iran	4,728	164	3.5	NR	NR	5
Mostafavi et al. (57)	2017	Razavi Khorasan	2,604	514	19.7	22.4	18.1	6
Akbarzadeh et al. (58)	2019	Fars	9,264	919	9.9	7.6	11.9	7
Alizadeh et al. (59)	2022	Kerman	600	106	17.7	6.8	10.8	5

QA, Quality assessment; NR, No Report.

### 3.3 Risk of bias in studies

Fifty studies earned a score of 4–7 (moderate risk of bias) based on the quality evaluation score, whereas three studies received an 8–9 (low risk of bias) score (Table 1).

### 3.4 Results of syntheses

**Figure 2:**
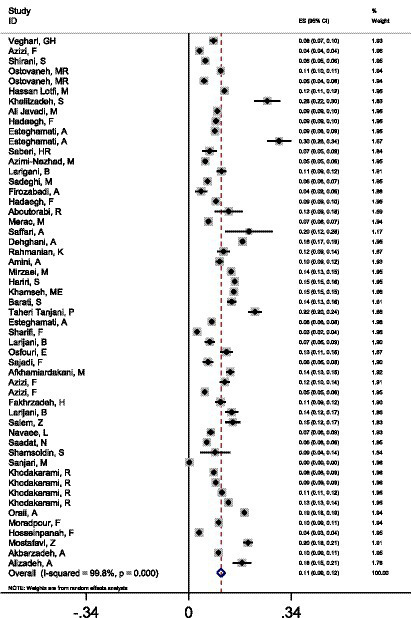
Forest plot analysis of diabetes prevalence in Iran, 1998–2023.

The study includes 53 studies with a total sample size of 1,244,896, of whom 89,979 tested positive for type 2 diabetes. Figure 2 shows that 10.80% (95% CI: 9.05–12.20%) of people have type 2 diabetes. However, there was a lot of statistical heterogeneity among the studies (*I*^2^ = 99.8%, Q statistic = 34073.31, df = 52, *p* < 0.0001, Tau-squared = 0.0024). By eliminating the one-by-one studies technique, we carried out a full sensitivity analysis, and the results showed that no one research had a substantial influence on the prevalence of diabetes; hence, the analysis did not allow us to discover any important studies in this regard (Table 2).

**Table 2:** Sensitivity analysis by removing one-by-one studies.

Reference ID	%(95% CI)
Omitting Veghari et al. (13)	10.85 (9.04–12.21)
Omitting Azizi et al. (14)	10.97 (9.05–12.44)
Omitting Shirani et al. (15)	10.91 (9.05–12.28)
Omitting Ostovaneh et al. (16)	10.81 (9.04–12.16)
Omitting Ostovaneh et al. (16)	10.91 (9.05–12.27)
Omitting Lotfi et al. (17)	10.78 (9.04–12.13)
Omitting Khalilzadeh et al. (9)	10.55 (9.02–11.90)
Omitting Ali et al. (18)	10.83 (9.04–12.19)
Omitting Hadaegh et al. (19)	10.84 (9.04–12.20)
Omitting Esteghamati et al. (20)	10.85 (9.04–12.20)
Omitting Esteghamati et al. (21)	10.48 (9.01–11.83)
Omitting Saberi et al. (22)	10.87 (9.05–12.23)
Omitting Azimi-Nezhad et al. (23)	10.91 (9.05–12.27)
Omitting Larigani et al. (24)	10.80 (9.04–12.16)
Omitting Sadeghi et al. (25)	10.90 (9.05–12.27)
Omitting Firozabadi et al. (26)	10.93 (9.05–12.29)
Omitting Hadaegh et al. (27)	10.84 (9.04–12.20)
Omitting Aboutorabi et al. (28)	10.76 (9.04–12.12)
Omitting Meraci et al. (29)	10.89 (9.05–12.25)
Omitting Saffari et al. (30)	10.69 (9.03–12.04)
Omitting Dehghani et al. (31)	10.66 (9.03–12.00)
Omitting Rahmanian et al. (32)	10.79 (9.04–12.14)
Omitting Amini et al. (33)	10.81 (9.04–12.17)
Omitting Mirzaei et al. (34)	10.74 (9.03–12.08)
Omitting Hariri et al. (35)	10.71 (9.03–12.03)
Omitting Khamseh et al. (36)	10.66 (9.05–11.80)
Omitting Barati et al. (37)	10.73 (9.03–12.09)
Omitting Tanjani et al. (38)	10.59 (9.02–11.94)
Omitting Esteghamati et al. (39)	10.88 (9.04–12.31)
Omitting Sharifi et al. (40)	10.96 (9.06–12.32)
Omitting Larijani et al. (41)	10.88 (9.05–12.23)
Omitting Osfouri (60)	10.77 (9.04–12.12)
Omitting Sajadi (61)	10.89 (9.05–12.24)
Omitting Afkhamiardakani et al. (43)	10.74 (9.03–12.10)
Omitting Azizi et al. (44)	10.78 (9.04–12.14)
Omitting Azizi et al. (45)	10.91 (9.05–12.28)
Fakhrzadeh et al. (46)	10.81 (9.04–12.16)
Omitting Larijani et al. (41)	10.74 (9.03–12.09)
Omitting Salem et al. (48)	10.73 (9.03–12.09)
Omitting Navaee et al. (49)	10.87 (9.05–12.23)
Omitting Saadat et al. (50)	10.90 (9.05–12.27)
Omitting Shamsoldin et al. (51)	10.83 (9.04–12.19)
Omitting Sanjari et al. (52)	11.0 (9.06–12.41)
Omitting Khodakarami et al. (53)	10.86 (9.04–12.28)
Omitting Khodakarami et al. (53)	10.84 (9.04–12.21)
Omitting Khodakarami et al. (53)	10.80 (9.04–12.15)
Omitting Khodakarami et al. (53)	10.75 (9.04–12.09)
Omitting Oraii et al. (54)	10.65 (9.03–11.99)
Omitting Moradpour et al. (55)	10.82 (9.04–12.18)
Omitting Hosseinpanah et al. (56)	10.95 (9.05–12.32)
Omitting Mostafavi et al. (57)	10.63 (9.03–11.98)
Omitting Akbarzadeh et al. (58)	10.82 (9.04–12.18)
Omitting Alizadeh et al. (59)	10.68 (9.03–12.03)

Additionally, the prevalence of diabetes was 14.4% (95% CI: 12.2–16.7) for people aged 45 to 55, 21.7% (95% CI: 17.5–25.0) for people aged 55 to 65, and 21.2% (95% CI: 10.7–31.7) for those aged 65 and above.

The prevalence of diabetes was also found to be related with obesity, with estimates of 8.4% (95% CI: 5–11.7), 12.4% (95% CI: 8.04–16.4), 17.4% (95% CI: 11.3–23.6), and 19% (95% CI: 7.2–30.2) in adults with a BMI of 18.5–24.9, 25–29.9, 30–34.9, and 35–39.9, respectively.

Moreover, there has been a notable rise in the incidence of diabetes throughout the last quarter-century. The prevalence of diabetes was assessed for five different time periods: 1988–2002, 2003–2007, 2008–2012, 2013–2017, and 2018–2023. The estimated prevalence rates for these years were 7.08% (95% CI: 5.09–9.06), 9.05% (95% CI: 7.08–11.10), 9.14% (95% CI: 8.36–9.91), 15.0% (95% CI: 11.40–18.60), and 13.40% (95% CI: 11.40–15.30), respectively (Table 3).

**Table 3:** Subgroup analysis of the prevalence of Diabetes in Iran, 1998–2023.

Category	No. studies	No. examined	No. positive	Prevalence% (95%CI)	Heterogeneity			
					*χ* ^2^	*p*-value	*I*^2^ (%)	Tau-squared
*Gender*								
Male	37	388,939	28,075	10.80%(9.1–12.4)	7191.56	<0.001	99.5%	0.0024
Female	35	515,180	50,352	13.4%(11.6–15.3)	8371.21	<0.001	99.6%	0.0030
*Age group*								
25–34	15	71,482	2,656	3.0%(2.0–3.09)	788.42	<0.001	98.2%	0.0003
35–44	16	32,930	3,006	7.02 (4.02–9.05)	1102.69	<0.001	99.3%	0.0022
45–54	19	17,763	3,086	14.4%(12.2–16.7)	3043.83	<0.001	99.4%	0.0024
55–64	19	19,034	5,039	21.7%(18.5–25.0)	1212.71	<0.001	98.5%	0.0049
≥65	4	3,325	804	21.2%(10.7–31.7)	149.92	<0.001	98.0%	0.0108
*Place*								
Urban	10	1,808,338	8,665	7.0%(6.7–7.4)	7348.31	<0.001	99.9%	0.00001
Rural	8	515,524	2054	3%(2.5–3.1)	1820.29	<0.001	99.6%	0.00001
*BMI*								
<18.5	3	1,080	20	1.9%(0.04–3.04)	4.23	0.120	52.8%	0.0001
18.5–24.9	9	23,607	1925	8.4%(5–11.7)	1279.74	<0.001	99.4%	0.0025
25–29.9	9	26,472	4,075	12.4%(8.04–16.4)	696.99	<0.001	98.9%	0.0037
30–34.9	9	19,072	3,917	17.4%(11.3–23.6)	961.68	<0.001	99.2%	0.0087
35–39.9	4	1,165	187	19%(7.2–30.2)	3.87	0.049	74.2%	0.0054
*Year*								
1998–2002	10	26,715	1877	7.08%(5.09–9.06)	301.10	<0.001	97.0%	0.0008
2003–2007	11	613,918	22,903	9.05%(7.08–11.10)	2381.97	<0.001	99.6%	0.0007
2008–2012	12	155,799	11,952	9.14%(8.36–9.91)	277.14	<0.001	96.0%	0.0002
2013–2017	7	28,311	2068	15.0%(11.40–18.60)	428.45	<0.001	98.6%	0.0022
2018–2023	13	400,153	51,179	13.40 (11.40–15.30)	3876.35	<0.001	99.7%	0.0013

**Figure 3:**
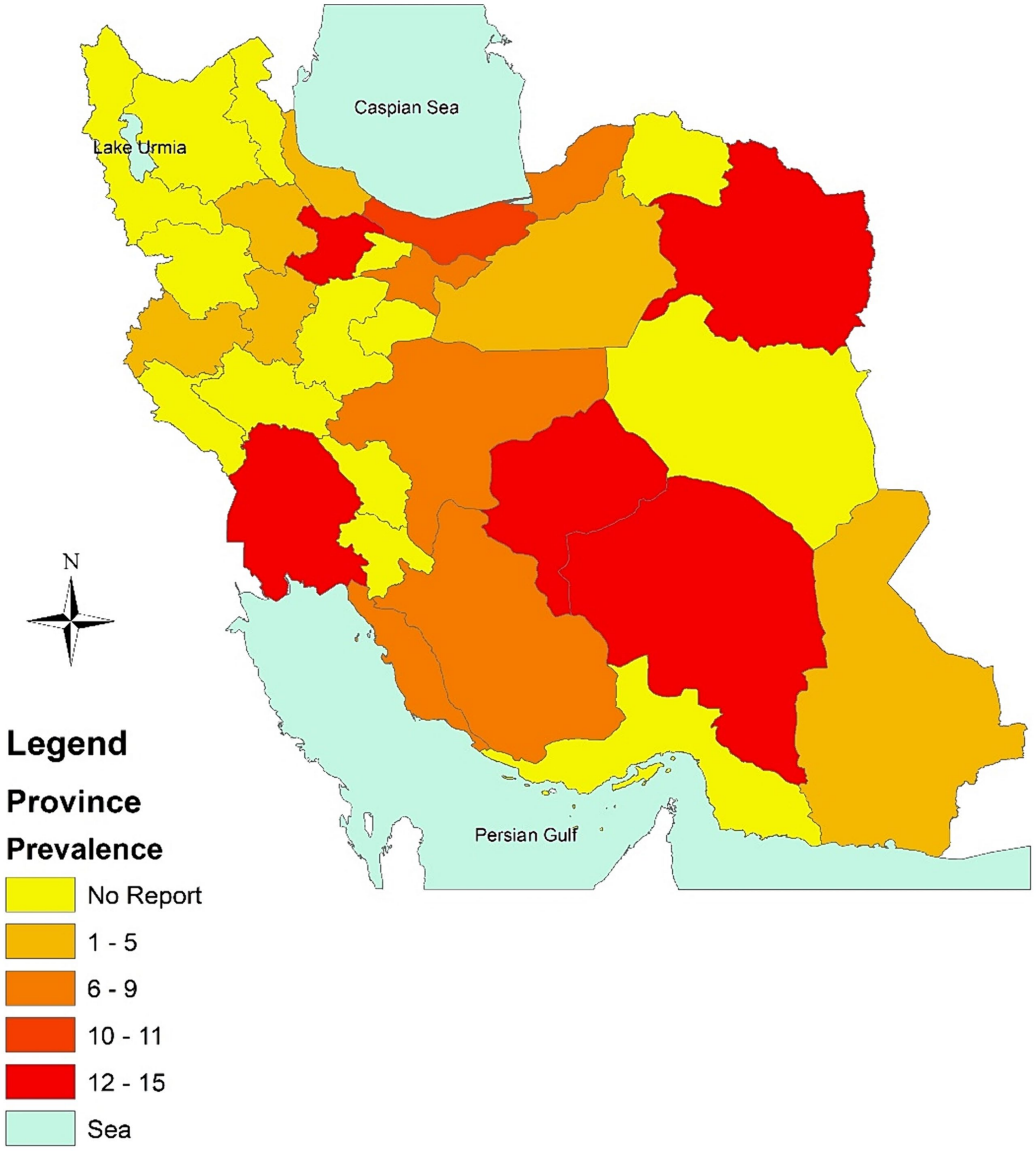
Prevalence of diabetes in Iran, 1998–2023 based on Province.

According to the findings of the subgroup analysis, it was seen that Khuzestan exhibited the greatest prevalence of type 2 diabetes, with a rate of 15.3%. This was closely followed by Razavi Khorasan, Qazvin, and Yazd, which reported rates of 14.4, 14.3, and 12.6%, respectively. The province of Mazandaran had a somewhat reduced incidence rate of 10.7%. In contrast, the prevalence rates of type 2 diabetes were comparatively lower in Fars (8.6%), Golestan (8.3%), Bushehr (8.1%), and Tehran (7.5%) among the population under study (Figure 3).

Univariate meta-regression was used to look into the causes of heterogeneity. According to the meta-regression analysis’s findings, a number of factors, including province (*B* = –0.005, P0.001), year (*B* = 0.0050, *p* = 0.020), gender (*B* = 0.0060, *p* = 0.022), age group (*B* = 0.164, *p* = 0.039), and BMI (*B* = 0.1174, *p* = 0.017), may be sources of heterogeneity (Table 4). The findings of the subgroup analysis showed that the prevalence of diabetes was 13.4% in women and 10.80% in men (95% confidence interval [CI]: 9.1–12.4).

**Table 4:** Results of meta-regression analyses for identifying potential contributors to heterogeneity among studies in the meta-analysis.

Possible cause of heterogeneity	Univariate	
	Coefficient (95%CI)	*p*-value
Province	−0.0005(−0.0006, −0.0005)	<0.001
Risk of bias	0.0220(−0.0060, 0.0501)	0.121
Year	0.0050 (0.0008, 0.009)	0.020
Gender	0.00601 (0.0008, 0.0091)	0.022
Age group	0.1644 (0.008, 0.3201)	0.039
Sample size	−0.000022 (0.000008,0.0000439)	0.499
BMI	0.1174 (0.0512, 0.2724)	0.017
Place(Urban/Rural)	−0.0261(−0.1562, 0.1025)	0.685

**Figure 4:**
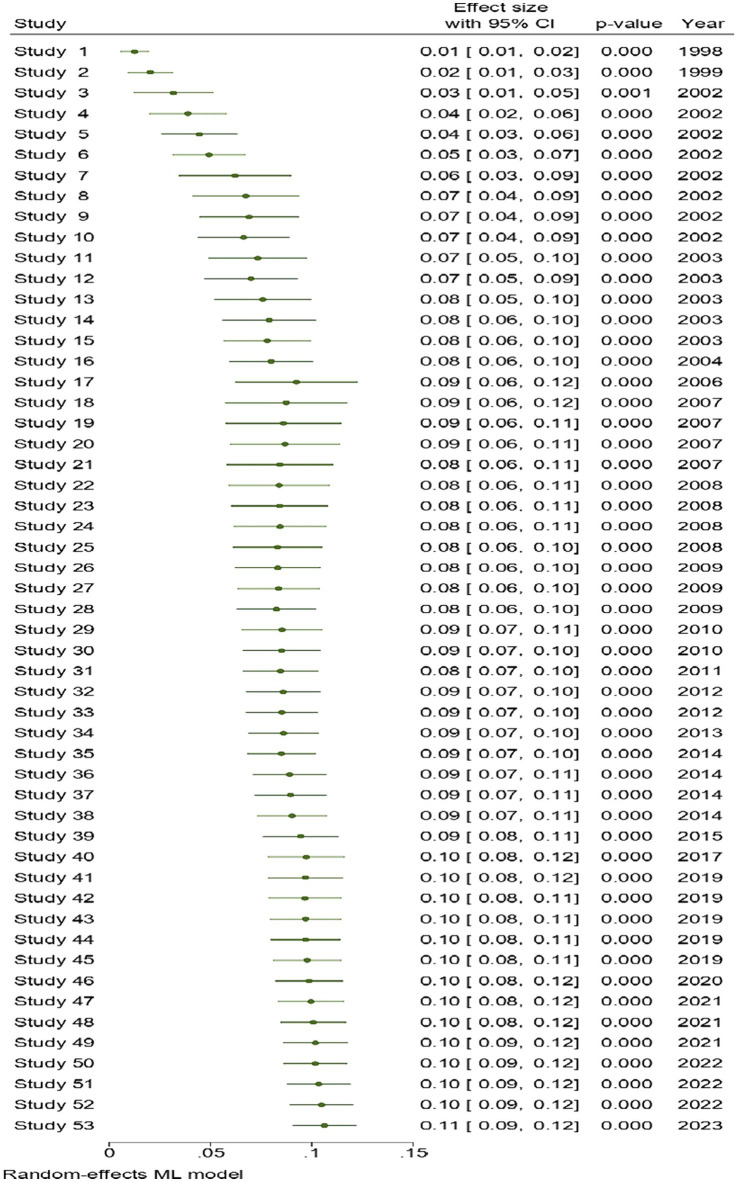
Cumulative meta-analysis results for examining the trend of changes in diabetes prevalence over time.

In addition, the use of cumulative meta-analysis was applied to examine the pattern of changes in the prevalence of diabetes over a certain period. The findings of the cumulative meta-analysis demonstrate a progressive rise in the incidence of diabetes over a period of time (Figure 4).

### 3.5 Reporting publication biases

**Figure. 5:**
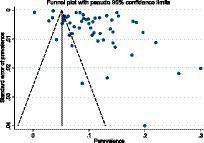
Funnel plot with pseudo 95% confidence interval limits for the detection of publication bias.

Significant publication bias was found in the articles included in this meta-analysis, according to the findings of Egger’s regression test and asymmetry in the funnel plot (bias = 14.65, 95% CI: 7.37–21.92, *p* = 0.001) (Figure 5). This bias was corrected using a non-parametric Trim-and-fill model, which predicted that three potential studies on the prevalence of diabetes were left out of the meta-analysis. The estimated pooled prevalence of diabetes using this model, adjusted for random effects, was 10.2% (95% CI: 8.09–11.5) (Table 5).

**Table 5:** Comparison of common and corrected meta-analysis results for publication bias.

Type of meta-analysis	Method	Number of studies	Pooled prevalence (95% CI)
Usual meta-analysis	Random-effects	53	10.80% (9.05–10.30)
Filled meta-analysis	Random-effects	56	10.20% (8.09–11.15)

## 4 Discussion

Between 1996 and 2023, every published study on the prevalence of type 2 diabetes among people in Iran was comprehensively reviewed. In this research, an effort was undertaken to extract the updated prevalence till 2023 for use by policymakers since a meta-analysis had previously published the pooled prevalence of diabetes from 1996 to 2004 (8).

The combined prevalence of diabetes among Iranians over 25 years old was 10.80% (95% CI: 9.05–12.20%), according to the findings of the current research. In other words, 1 in 10 adults over the age of 25 has diabetes. This figure was lower than that of Pakistan (14.62%) (62) and Afghanistan (12.13%) (63), two adjacent nations with comparable cultures, and higher than that of Nepal (8.4%) (64) and Bangladesh (7.8%) (65) in Africa. The lower age limit of participants in Nepal and Bangladesh (over 15 in Nepal and over 20 in Bangladesh) may partially account for the higher aggregated prevalence in Iran compared to Nepal and Bangladesh. However, the reasons for the lower prevalence of diabetes in Iran compared to Pakistan and Afghanistan with a comparable age range are unclear and may be attributable to differences in the age distribution, demographic characteristics, and lifestyle of the population. It is crucial to acknowledge that the observed differences in prevalence among these countries might be influenced by various factors, including differences in study methodologies, sample sizes, and population demographics. Therefore, a more detailed examination of these factors is needed to establish robust comparisons. Future research could delve deeper into the specific methodologies employed in each country, diagnostic criteria, and sampling techniques, as well as considering the age distribution and lifestyle factors, to provide a more comprehensive understanding of the observed variations.

The prevalence of type 2 diabetes in Iran was estimated to be 9.34% based on individual studies published between 1996 and 2004 and in persons over the age of 18 according to a meta-analysis (8). As an addendum to the aforementioned study, the prevalence of diabetes in adults aged 25 and older was estimated to be 10.8% in the present study. Moreover, subgroup analysis by publication year revealed that the five-year prevalence of diabetes was 7.08 (1998–2002), 9.05 (2003–2007), 9.14 (2008–2012), 15, and 13.4 (2018–2023). Upon closer analysis, we identified several factors that may contribute to the observed increase. Firstly, our data encompass various time periods, each representing distinct stages of diabetes awareness, screening, and diagnostic capabilities in Iran. The initiation of the National Diabetes Screening Program in 2006, coupled with advancements in detection and diagnosis, may have led to an increased identification of previously undiagnosed cases. To provide a more comprehensive perspective, the findings of a 2022 study based on data from the four periods of the national STEPs study, the prevalence of diabetes among adults aged 25–65 in 2004, 2007, 2011, and 2016 was 8.4, 11.1, and 13.2 percent, respectively (53). Consequently, it appears that the prevalence of diabetes in Iran has fluctuated over the past few years. The factors behind the rise of diabetes throughout time are complicated and multifaceted. One of them is the aging population, and as people live longer and age, diabetes will certainly afflict them more frequently (66). Additionally, lifestyle changes and an aging population are acknowledged contributors to the rising prevalence of diabetes. As people live longer and age, the likelihood of developing diabetes increases. According to some data, Iran’s population of people aged 60 and older will reach 20% in 2050 (67), exceeding 10% in 2022 (67). Moreover, the Iranian population has experienced an upsurge in diabetes risk factors such as obesity, sedentary behavior, and unhealthy dietary patterns (68–70).

As a result of steady advancements in diabetes detection and diagnosis over the years in Iran, the percentage of undiagnosed diabetes decreased from 45.7 to 24.7% during a period of seven years (71). As a result, it seems that the observed increase in the cumulative prevalence of diabetes over time in the current research is rather accurate.

The subgroup analysis reveals significant differences in the prevalence rates among provinces, with Khuzestan exhibiting the highest rate at 15.3%. The closely following rates in Razavi Khorasan, Qazvin, and Yazd, as well as the comparatively lower rates in Mazandaran, Fars, Golestan, Bushehr, and Tehran, offer insights into the geographical distribution of type 2 diabetes in Iran. One plausible explanation for the disparities in diabetes prevalence among provinces could be lifestyle differences. Variations in dietary habits, physical activity levels, and cultural practices may influence the prevalence of risk factors for type 2 diabetes. Future studies exploring the relationship between lifestyle factors and diabetes prevalence in specific regions could provide valuable insights. The socio-economic landscape of each province may also play a pivotal role. Disparities in income, education levels, and access to healthcare services can contribute to variations in diabetes prevalence. Furthermore, Differences in regional health policies and healthcare infrastructure might contribute to variations in diabetes prevalence. Assessing the effectiveness of existing health programs, accessibility of healthcare services, and the implementation of preventive measures could shed light on the impact of regional health policies on diabetes prevalence.

The subgroup analysis revealed an association between overweight obesity and diabetes. The prevalence of diabetes increases dramatically as body mass index (BMI) rises, so the prevalence of diabetes among those with a BMI between 35 and 39.9 was ten times that of those with a BMI below 18.5. The research performed on the association between diabetes and obesity demonstrates that overweight and obesity, especially when accompanied by abdominal obesity, is a significant risk factor for diabetes and prediabetes due to beta cell dysfunction and insulin resistance via three mechanisms (72): Increase in the production of cytokines such as tumor necrosis factor-alpha, resulting in a decrease in adiponectin (73); Misplaced deposition of lipids in the liver (74); and Mislocalization of lipids in the available research indicates that there has been a notable rise in the prevalence of obesity in Iran from 1990 to 2016, with a threefold increase seen over this time span. Furthermore, concomitant with the rise in population size and the increase in body mass index (BMI), there has been a nearly 11-fold surge in the prevalence of obesity throughout this timeframe (68). Furthermore, the findings of the STEPS research, which was done in 2016 on individuals aged 18 and above at a national level, yielded an estimated prevalence of 36.6% for overweight and 22.7% for obesity (75). In the meanwhile, a meta-analysis on the prevalence of overweight and obesity in Middle Eastern nations from 2000 to 2020 found that the prevalence of overweight was 33.92, and the prevalence of obesity was 22.41 (76). Adequate policies should be established to minimize these variables in the Iranian population to lower the incidence of type 2 diabetes, given the substantial prevalence and trend of overweight and obesity in Iran as a key risk factor for the disease. Implementing a robust public health strategy is essential to combat the escalating prevalence of obesity and mitigate the associated risks, particularly in reducing the incidence of type 2 diabetes among the Iranian population. This multifaceted approach includes launching culturally relevant public awareness campaigns to educate the public about the risks of obesity and its direct link to diabetes. Simultaneously, comprehensive and accessible nutritional education programs are crucial, focusing on balanced nutrition and the benefits of a healthy diet. To counteract sedentary lifestyles, initiatives promoting regular physical activity at community and workplace levels should be developed and incentivized. Widespread access to healthcare services, with a focus on preventative care for early detection and management of obesity-related conditions, is paramount. Advocacy for evidence-based policies, including regulations on food marketing, urban planning encouraging physical activity, and workplace wellness programs, is essential.

According to the findings of the current research, those aged 55 to 64 had a seven-fold higher incidence of diabetes than those aged 25 to 34. The age-specific prevalence in the 55 and older age group was found to be more than double that of those between the ages of 25 and 34 in a meta-analysis carried out in Afghanistan (63), a country next to Iran. Another Malaysian meta-analysis found that adults 60 years and older had a greater than tenfold increased risk of developing diabetes than those between the ages of 20 and 29 (77). There is no proof that the association between age and diabetes is inverse or nonexistent. As a result, it seems that the prevalence of diabetes rises with age across the board (78). Age-related physiological changes may have an impact on the prevalence of diabetes. Diabetes rates rise as people become older because of insulin resistance in the skeletal muscles brought on by changes in adiposity and physical inactivity on the one hand (79) and reduced secretory capacity and loss or malfunction of pancreatic beta cells on the other (80). Moreover, acknowledging the potential influence of lifestyle factors and healthcare access in different age groups is essential. Exploring the impact of dietary habits, physical activity levels, and healthcare-seeking behavior on diabetes prevalence within distinct age brackets would offer valuable insights.

In this research, women had a greater prevalence of diabetes [13.4 percent (11.6–15.3)] than males (10.80%, 9.1–12.4). Similar studies carried out in nearby nations and nations sharing a similar culture revealed that the prevalence of diabetes was higher in women than in men in some nations, including Turkey (81), Pakistan (62), and Nepal (64), while in Bangladesh the prevalence was slightly higher in men than in women (65). There was no distinction between the sexes in Cameron (82). Although women were more likely to have diabetes than males in the majority of the studies included in this meta-analysis, the situation was reversed in six of them. There may be a number of reasons for the varied frequency in the two genders. Estrogen, a female hormone important for insulin sensitivity and glucose metabolism, is one of these elements. As a result, low estrogen levels, particularly during menopause, might raise insulin resistance and diabetes risk (78). Obesity and way of life are other variables that determine the differing prevalence between the sexes. A Middle Eastern meta-analysis found that women are more likely than males to suffer from general obesity, which is a significant risk factor for developing diabetes (76). Stress, physical inactivity, and a diet high in processed foods and sugar are additional risk factors for type 2 diabetes that are more common in women (83). However, males are more likely to develop visceral fat buildup and abdominal obesity, both of which are risk factors for diabetes (84). In addition to the above indicated elements, gender differences exist in healthcare seeking behavior. Relevant research have shown that males often postpone going to the doctor when they are ill and use the less extensive array of healthcare services (85). Women are more likely than males to seek medical attention when they are unwell, which increases the likelihood that their condition will be discovered (86, 87). Moreover, the impact of cultural expectations and societal norms on gender-specific health behaviors cannot be overlooked. Societal pressures may influence both men and women in distinct ways, affecting their dietary choices, stress levels, and propensity for physical activity. Understanding these cultural dynamics is vital for tailoring effective public health interventions. Additionally, healthcare-seeking behavior disparities between genders may be shaped by socio-cultural factors. Delving into the reasons behind delayed medical visits among men and the more proactive approach of women toward healthcare utilization can provide valuable insights. Cultural perceptions of health, gender roles, and accessibility to healthcare services play pivotal roles in shaping these behaviors. A comprehensive exploration of the synergistic effects of lifestyle, obesity, and healthcare-seeking behavior, embedded within the broader context of cultural and societal influences, will contribute to a more nuanced understanding of gender disparities in diabetes prevalence.

## 5 Strengths

The following are some of this study’s advantages: First of all, the research includes a large number of studies, totaling 1,244,896 people in the sample. The reliability and generalizability of the results are greatly improved by the size of the sample. Secondly, by expanding the analysis to encompass data through 2023, the research provides a critical update to a prior meta-analysis. More updated information on the incidence of type 2 diabetes in Iran is provided by this extension. Last but not least, using cumulative meta-analysis enables tracing the historical trend in diabetes prevalence, giving important historical context.

## 6 Limitations

The limitations of the current meta-analysis should be taken into account when interpreting the findings. First, age-specific prevalence was not reported in two-thirds of the papers that made up this meta-analysis. If the contribution of participants under 40 years old in these studies is significant, the prevalence of diabetes in the present study may have been underestimated. The prevalence of diabetes is typically 5–10% in people under the age of 40, and it rises sharply from that age and above. Second, several studies failed to indicate whether individuals with diabetes were diagnosed with type 2 or another kind. Since type 2 diabetes affects more than 90% of diabetes patients, the analyzed condition was referred to as type 2 diabetes in the publications that were included. Third, since there was a significant level of heterogeneity in the current analysis, 99.8 of the variations in the prevalence estimate were due to the heterogeneity across studies rather than chance. Although we used meta-regression to track out its source and subgroup analysis to somewhat lessen its impact, it may still have an impact on the study’s overall estimate and conclusions.

## 7 Conclusion

In conclusion, our comprehensive meta-analysis spanning the period from 1996 to 2023 reveals a noteworthy estimated prevalence of 10.8% for type 2 diabetes in Iran. This alarming prevalence underscores the urgency of addressing this public health challenge. The observed variations across gender, age groups, and geographic regions emphasize the need for nuanced, targeted interventions. Our findings call for the implementation of proactive measures, including tailored screening programs and lifestyle modification initiatives. Policymakers and healthcare practitioners should take heed of the temporal trends, especially the significant increase from 2013 to 2017, prompting the development of effective strategies to mitigate the future burden on the healthcare system.

## Data availability statement

The datasets presented in this study can be found in online repositories. The names of the repository/repositories and accession number (s) can be found in the article/Supplementary material.

## Ethics statement

We diligently adhered to ethical principles for our systematic review and meta-analysis studies throughout this research. The study protocol obtained official approval from the Ethics Committee of Torbat Jam Faculty of Medical Sciences, with the assigned code IR.TRJUMS.REC.1401.004.

## Author contributions

NH: Formal analysis, Investigation, Methodology, Project administration, Validation, Visualization, Writing – original draft, Writing – review & editing. MJ: Conceptualization, Data curation, Formal analysis, Investigation, Methodology, Software, Validation, Writing – original draft. NN: Data curation, Investigation, Methodology, Writing – original draft. SH: Data curation, Investigation, Methodology, Software, Writing – original draft. VR: Conceptualization, Data curation, Formal analysis, Investigation, Methodology, Project administration, Software, Supervision, Validation, Visualization, Writing – original draft, Writing – review & editing.
