# Supplementary material for: An updated systematic review and Meta-analysis of the prevalence of type 2 diabetes in Iran, 1996–2023
Source: Front Public Health. 2024 Apr 4;12:1322072. doi: 10.3389/fpubh.2024.1322072 (PMC11025666; doi:10.3389/fpubh.2024.1322072)
Supplement: Supplementary file 1 [file Table_1.DOCX]

S1 Table: Strategy search for the Prevalence of type 2 diabetes in Iran

| **Databases** | **Search terms** |
| --- | --- |
| Google Scholar, Scopus, PubMed/MEDLINE, Science Direct, Web of Science, EMBASE, Science Direct, ProQuest  SID (Scientific Information Database), Magiran, element, | (diabetes mellitus) OR (hyperglycemia) OR (Non-Insulin-Dependent Diabetes Mellitus) OR (Diabetes Mellitus, Type II) OR (Type 2 Diabetes) OR (Diabetes, Type 2) OR (NIDDM) OR (Stable Diabetes Mellitus) OR (Ketosis-Resistant Diabetes Mellitus), in addition to the keywords "prevalence" or "epidemiology" and "Iran." |
| **Number of papers identified** | **Results in each database** |
| 2860 Journal papers | Google Scholar=571 papers  PubMed=458 papers  Scopus=422 papers  Embase=388 papers  Web of Science=262 papers  Science Direct=248 papers  ProQuest=135 papers  Magiran= 180 papers  SID=110 papers  Element=86 papers |
